# Identification of Nitrogen Starvation-Responsive MicroRNAs in *Arabidopsis thaliana*

**DOI:** 10.1371/journal.pone.0048951

**Published:** 2012-11-14

**Authors:** Gang Liang, Hua He, Diqiu Yu

**Affiliations:** 1 Key Laboratory of Tropical Forest Ecology, Xishuangbanna Tropical Botanical Garden, Chinese Academy of Sciences, Kunming, Yunnan, China; 2 The Graduate School of the Chinese Academy of Sciences, Beijing, China; French National Center for Scientific Research - Institut de biologie moléculaire et cellulaire, France

## Abstract

microRNAs (miRNAs) are a class of negative regulators that take part in many processes such as growth and development, stress responses, and metabolism in plants. Recently, miRNAs were shown to function in plant nutrient metabolism. Moreover, several miRNAs were identified in the response to nitrogen (N) deficiency. To investigate the functions of other miRNAs in N deficiency, deep sequencing technology was used to detect the expression of small RNAs under N-sufficient and -deficient conditions. The results showed that members from the same miRNA families displayed differential expression in response to N deficiency. Upon N starvation, the expression of miR169, miR171, miR395, miR397, miR398, miR399, miR408, miR827, and miR857 was repressed, whereas those of miR160, miR780, miR826, miR842, and miR846 were induced. miR826, a newly identified N-starvation-induced miRNA, was found to target the *AOP2* gene. Among these N-starvation-responsive miRNAs, several were involved in cross-talk among responses to different nutrient (N, P, S, Cu) deficiencies. miR160, miR167, and miR171 could be responsible for the development of *Arabidopsis* root systems under N-starvation conditions. In addition, twenty novel miRNAs were identified and nine of them were significantly responsive to N-starvation. This study represents comprehensive expression profiling of N-starvation-responsive miRNAs and advances our understanding of the regulation of N homeostasis mediated by miRNAs.

## Introduction

Nitrogen (N) is one of the most important macronutrients essential for plant growth and development. N accounts for 1.5–2.0% of plant dry matter and is required for the synthesis of proteins, nucleotide acids, chlorophylls, and so on [1]. To grow and develop normally, plants must obtain sufficient N from the soil via their roots. However, there is not always sufficient N in the soil to meet the N requirements of plants, because soil N levels are affected by many factors such as soil erosion, rainwater leaching, and microbial consumption. Plants have already evolved a number of ways to conserve and mobilize internal N and to increase acquisition of external N. To adapt to N-limited conditions, plants must sense changes in internal and external N concentrations. The sensing and signal transduction networks that control plant responses to N deficiency are not well characterized.

Recent studies have revealed that plant small RNAs play a pivotal role in regulating gene expression at post-transcription levels. microRNAs (miRNAs), a class of non-coding small RNAs, originate from stem-loop structures of primary transcripts. They are processed into precursor RNA and then mature miRNAs 20–21 nt in length via the activities of Dicer-like proteins in the nucleus [2]. HYL1 (HYPONASTIC LEAVES 1) and SE1 (SERRATE 1) ensure the accurate processing of miRNAs [3]. The processed miRNAs are further methylated at the 3′ terminal by HEN1 (HUA ENHANCER 1) [4] and exported from the nucleus into the cytoplasm where they are incorporated into the RNA-induced silencing complex (RISC). In the RISC, the miRNAs recognize their targets through complementary base pairing and cleave target transcripts or/and repress target translation [5]–[7].

Plant miRNAs have been found in diverse plant species, including monocotyledons (e.g. *Oryza sativa*, *Zea mays*, *Sorghum bicolor*), dicotyledons (e.g. *Arabidopsis thaliana*, *Medicago truncatula*, *Populus trichocarpa*), mosses [8], and unicellular algae [9]. Plant miRNAs are involved in the regulation of plant growth and development, biotic and abiotic stresses, metabolic pathways and so on. Recently, plant miRNAs were shown to function in sensing nutrient stresses. For example, miR395 is induced by sulfate starvation and regulates sulfate accumulation and allocation by targeting *APSs* (*ATP Sulfurylase*) and *SULTR2;1* (*SULFATE TRANSPORTER 2;1*), respectively [10]; miR399 is up-regulated by phosphate limitation and controls phosphate transport and redistribution by repressing *PHO2* (*PHOSPHATE 2*) [11]. In addition, by deep sequencing small RNAs, miR778, miR827, miR828, and miR2111 were also identified as being responsive to phosphate starvation [12], [13]. The homeostasis of another essential nutrient, copper, is regulated by miR398, which directs the degradation of *Copper/zinc Superoxide Dismutase* mRNA when copper is limited [14]. miR397, miR408, and miR857 also mediate the regulation of copper homeostasis by targeting several *Laccase* genes [15]. miR169 plays an important role in regulating nodule development in *M. truncatula* and its over-expression leads to decreased expression of the *MtHAP2-1* (*HAPLESS 2-1*) gene and a deficient N-fixation phenotype [16]. Meanwhile, over-expression of miR169 impairs the N-uptake system, leading to low N accumulation in *Arabidopsis* [17].

To date, however, there have been few studies on miRNA(s) involved in the N-deficiency response. It is unknown whether some miRNAs are specifically responsive to N limitation in *Arabidopsis*. Because of the vast dynamic range of miRNA expression, it is difficult to discover miRNAs expressed at low frequencies under normal growth conditions. Deep sequencing technologies can rapidly detect known and novel miRNAs with very high sensitivity [12], [13], [18], [19]. The aims of this study were to analyze the expression profiles of small RNAs in response to N deficiency and to identify novel miRNAs specifically induced by N deprivation. Therefore, Solexa sequencing technology was used to detect the small RNAs population in *Arabidopsis* grown under N-sufficient and N-deficient conditions. Our data provide insight into the authenticity of previously reported *Arabidopsis* miRNAs and identify some new miRNAs associated with N-starvation stress responses. It suggested that members from the same miRNA family showed differential accumulation in response to N starvation. Three conserved miRNAs were found to play roles in root system development under N starvation. Moreover, the finding that N starvation affects the expression of other nutrient stress-related miRNAs is intriguing, and adds new insight about how miRNAs regulate nutrient balance in plants.

## Results and Discussion

### Summary of small RNA profiles in response to N deficiency

To investigate the response of miRNAs to N deficiency, two small RNA libraries were constructed from N-sufficient (+N) and N-deficient (−N) seedlings by Solexa high-throughput sequencing technology. After adaptor trimming, the unique sequences were selected from the raw data and then mapped to the *Arabidopsis* genome. A total of ∼11 million raw reads were obtained from each library, and the clean reads after trimming adaptors accounted for ∼95%. Approximately 10 million sequence reads (89% and 91% of trimmed sequence reads in +N and −N libraries, respectively), corresponding to ∼1.5 million sequence signatures, mapped perfectly to the genome (The *Arabidopsis* Information Resource 8). The perfectly matched sequences were used for further analysis.

Most of the RNA sequences were within the range of 19 to 24 nt, which accounted for 90% of total clean reads. The correlation between the length of small RNAs and the proportion of total sequence reads was examined (Figure S1A). Two distinctive distributions were observed in +N and −N libraries. The library from the N-deficient samples showed fewer 24 nt sequences and more 20 nt sequences compared with those in the library from the N-sufficient samples. Subsequently, common and specific sequences were identified by comparing the two libraries. The +N-specific, −N-specific, and common sequences between the two libraries were 45.6, 38.95, and 15.99% respectively, for unique small RNAs, and 9.45, 7.98, and 82.57%, respectively, for total small RNAs (Figure S1B). The small RNAs were classified into rRNA, snRNA, snoRNA, tRNA, and other RNA according to their origin, and the proportion of each class was calculated (Table 1). A significant increase in small RNAs derived from tRNA was observed under N-deficient conditions; this trend was also observed under phosphate deficiency [12].

**Table 1:** Summary of small RNA sequencing data.

class	+N		−N	
	Unique sRNA	Total sRNA	Unique sRNA	Total sRNA
rRNA	18852(0.87%)	307668(3.12%)	16787(0.86%)	281621(2.73%)
snRNA	1116(0.05%)	2188(0.02%)	1907(0.10%)	5497(0.05%)
snoRNA	1668(0.08%)	3297(0.03%)	2209(0.11%)	6236(0.06%)
tRNA	6099(0.28%)	301115(3.11%)	6404(0.33%)	1007667(9.80%)
other	2131218(98.7%)	9067082(93.7%)	1915407(98.6%)	8984342(87.4%)
total	2158953	9681350	1942714	10285363

The small RNA sequences were used to analyze previously characterized miRNAs in *Arabidopsis* (miRBase release 14.0, www.miRbase.org). All sequences that matched perfectly to known miRNAs from miRbase were used for statistical analyses. A total of 186 miRNAs from the combined RNA sequence reads were identified. These miRNAs belonged to 98 miRNA families and represented 89% of all known unique *Arabidopsis* miRNA sequences and 83% of all known miRNA families (Table S1).

### Expression profiling of known miRNAs in response to N starvation

The small RNA sequences from the N-deficient and N-sufficient samples were analyzed for the presence of previously characterized miRNAs in *Arabidopsis*. The miR156 family was the most abundant (∼1,400,000 reads), although many miRNAs were expressed at low frequencies (read count fewer than 10).

Deep sequencing can distinguish and measure miRNA sequences with even one nucleotide change, which means that different members from the same miRNA family can be distinguished. Based on the sequencing results, different miRNA family members were expressed at vastly different frequencies. For example, miR158a and miR158b were represented by 331232 and 3383 reads respectively under N-sufficiency conditions, but 115256 and 2850 reads respectively under N-deficiency conditions (Table S2). Similar trends were observed for other miRNA families, such as miR156, miR169, and miR172.

**Figure 1:**
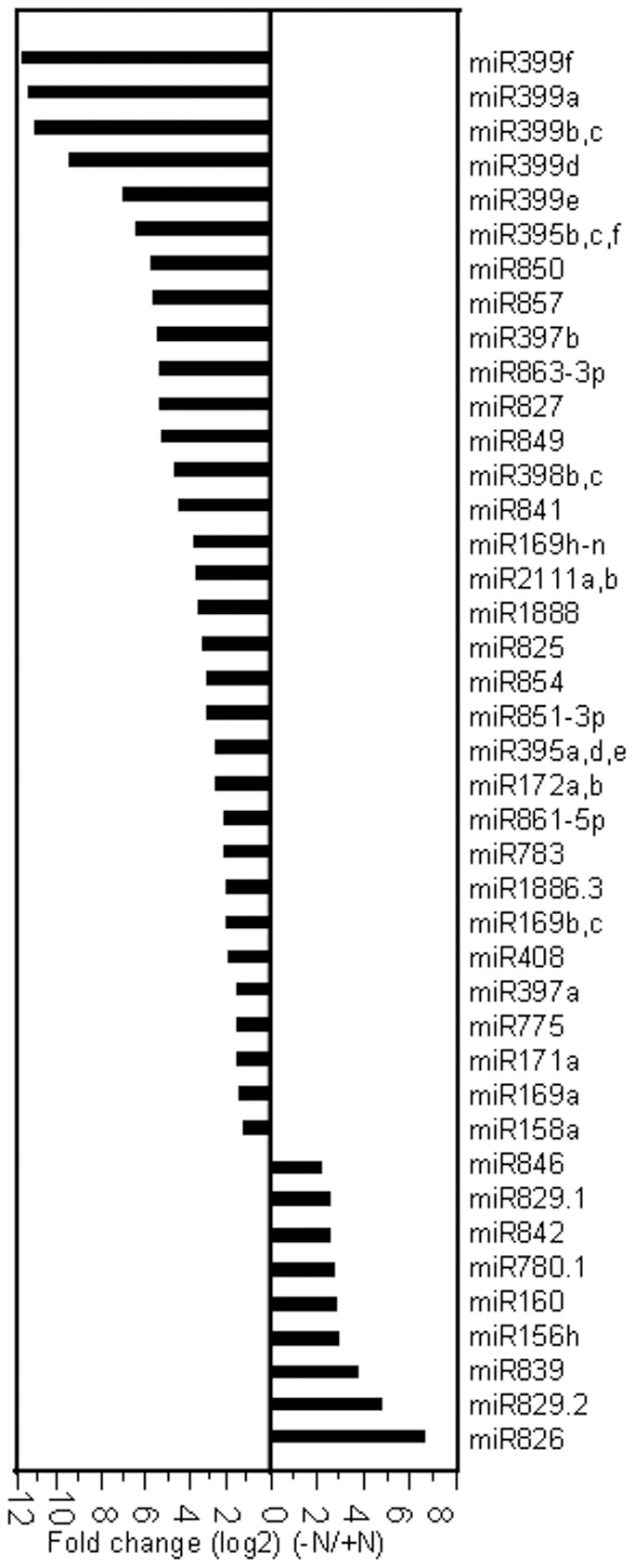
Differentially expressed miRNAs in response to N deficiency. The significantly differentially expressed miRNAs with greater than 3-fold relative change were shown.

Deep sequencing can also accurately quantify miRNA abundance. Among the miRNAs retrieved, the differentially expressed miRNAs with greater than 3-fold relative change in sequence count were identified (Figure 1). These miRNAs were defined as N-starvation-responsive miRNAs in the present study.

### Homologous miRNA members conversely respond to N starvation

**Figure 2:**
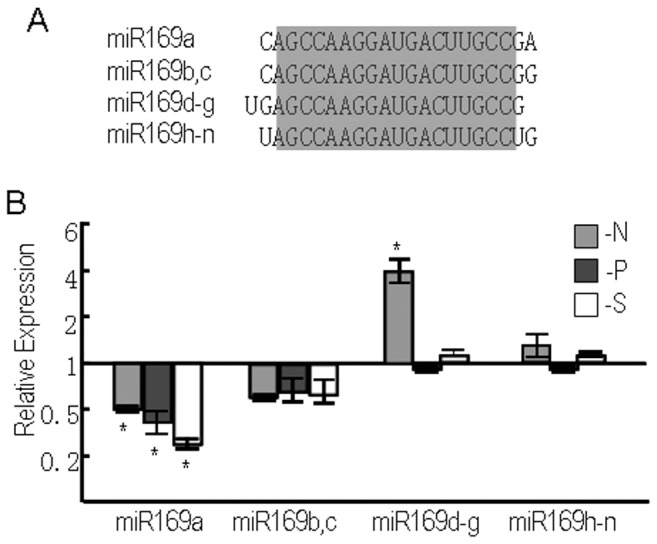
Differential expression of different miR169 species. (A) Four different mature miR169 species. (B) Expression of different miR169 members in response to different nutrient deficiencies. Gene expression values shown are relative to the expression in plants grown under normal MS medium, for which the value is set to 1. Error bars indicate ± SE obtained from three biological repeats. Values marked by an asterisk are significantly different from the corresponding control value with Student's *t*-test (*p*<0.01; *n* = 3).

Most conserved plant miRNA families contain more than two members. Recent studies revealed that ancient plant miRNAs originated from the inverted duplication of target gene sequences and some miRNA members are distributed in clusters. However, it is unclear whether different species of the same family have different functions. Over-expression of different miRNA species has resulted in very similar phenotypes, implying that all miRNA members can effectively suppress their target genes. Deep sequencing technology provides an approach to detect expression of different mature miRNAs. miR169 is a conserved plant miRNA that is found in diverse plant species. In *M. truncatula*, miR169 plays a role in regulating nodule development [16]. In *Arabidopsis*, N starvation decreased the expression of miR169. Plants overexpressing miR169 accumulated less N and were more sensitive to N starvation [17]. The *Arabidopsis* miR169 gene family contains 14 members, represented by four different mature miRNA species; miR169a, miR169bc, miR169d–g, and miR169h–n (Figure 2A). Unlike most miRNA families whose members show similar expression patterns, the different species of the miR169 family showed differential responses to N starvation (Table S2). The read count of the miR169d–g mature sequence increased under N deficiency, but those of the other species dramatically decreased. To confirm this result, stem-loop RT-PCR was used to determine the expression of four mature miR169s in the root under N, P, and S starvation (Figure 2B). Consistent with the sequencing results, miR169d–g, but not the other miR169 species, increased specifically upon N starvation. The differential expression implies that different miRNA species may have roles in specialized functions under N starvation conditions. Differential expression was also observed among members of the miR167, miR171, miR172, and miR319 families (Table S2). To understand the functions of different miRNA species, further research should focus on the spatial and temporal expression patterns of these miRNA species under N deficiency.

### Targets of N-starvation-responsive miRNAs

Under N-starvation conditions, miR399, miR395, miR850, miR857, miR863, and miR827 were significantly down-regulated, whereas miR160, miR826, miR839, and miR846 were dramatically up-regulated. These N-starvation-responsive miRNAs were further classified into two groups, N-starvation-suppressed (NSS) miRNAs and N-starvation-induced (NSI) miRNAs. To analyze the functions of these miRNAs, it is important to identify their targets, since plant miRNAs mainly negatively regulate expression of their target genes. Plant miRNAs recognize their target genes through complementary base-pairing, so it is easy to identify miRNA target genes by computational predictions. For miR156, miR160, miR169, miR171, miR172, miR395, miR397, miR398, miR399, miR408, miR775, miR780.1, miR827, miR842, miR846, miR857, and miR2111, their targets have been predicted and most of them were validated previously (Table 2). To identify candidate miRNA target genes of the other N-starvation-responsive miRNAs, the target prediction software package, WMD3 [20], was used. To improve the reliability of predicted targets, three mismatches or less for miRNA-target duplex was set. The potential targets were listed in the Table 2. No candidate targets were predicted for five miRNAs (miR829.1, miR839, miR850, miR851, and miR861). Although miR158 has been predicted to target a transcript encoding a pentatricopeptide (PPR) repeat-containing protein [21], we predicted two new targets of miR158, *At2g03210* and *At2g03220*, both of which encode fucosyltransferase proteins.

**Table 2:** N-starvation-responsive miRNAs and their targets.

Class	Families	Members	Target gene families	Target genes	Potential roles	Other nutrient stimuli	References
NSS	miR158	a	Pentatricopeptide repeat (PPR)	At1g64100			[21], [22]
			Fucosyltransferase	At2g03210	Glycosylation		
				At2g03220			
	miR169	a–c,h–n	CAAT binding factor	**At1g17590**	Nitrogen homeostasis	-P	[12], [17], [25], [61]
				**At1g54160**			
				**At1g72830+**			
				**At3g05690+**			
				**At3g20910**			
				**At5g06510+**			
				**At5g12840+**			
	miR172	a,b	AP2 transcription factor	**At5g60120**	Flower development		[58]–[60]
				**At4g36920**			
				**At2g28550**			
				**At2g28550**			
				**At5g67180**			
	miR395	a–f	ATP sulfurylase	**At3g22890+**	Sulfate homeostasis	-S -P	[10], [12], [57]
			Sulfate transporter	**At4g14680**			
				**At5g43780+**			
				**At5g10180**			
	miR397	a,b	Laccase	**At2g29130+**	Copper homeostasis	-Cu	[15], [56]
				**At2g38080+**			
				**At5g60020+**			
	miR398	b,c	Cu/Zn superoxide dismutase;	**At1g08830+**	Copper homeostasis	-Cu -P -K	[12], [15], [56]
			Cytochrome oxidase c;	**At2g28190+**			
			Copper chaperone	**At3g15640**			
				**At1g12520**			
	miR399	a–f	Ubiquitin conjugase E2	**At2g33770**+	Phosphate homeostasis	-P	[11]
	miR408		Laccase; Plantacyanin	**At2g30210+**	Copper homeostasis	-Cu	[15]
				**At5g05390−**			
				**At5g07130+**			
				**At2g02850+**			
	miR775		Fucosyltransferase	**At1g53290**	Glycosylation		[35]
	miR783		Protein of unknown	At4g01090	Unknown		
	miR825		*	*	*		
	miR827		E3 ligase with RING and SPX	**At1g02860+**	Nitrogen/Phosphorus metabolism	-P	[12], [27]
	miR841		Histone H2A	At2g38810	Unknown		[55]
				At4g13570			
	miR849		CXC domain-containing protein	At4g29000	Unknown		
	miR850		*	*	*		
	miR851	3p	*	*	*		
	miR854	a–d	Oligouridylate binding protein1b	**At1g17370**	Transcription regulation		[62]
	miR857		Laccase	**At3g09220+**	Copper homeostasis	-Cu	[15]
	miR861	5p	*	*	*		
	miR863	3p	Transducin/WD40 repeat-like other RNA	At2g40360+	rRNA process		[23], [25]
				At4g13495	Unknown		
	miR1886.3		Dentin sialophosphoprotein-related	At5g07970	Unknown		
	miR1888		Haloacid dehalogenase-like hydrolase	At5g65140	Trehalose biosynthesis		[22]
			SAUR-like auxin-responsive protein	At2g16580	Response to auxin stimulus		
	miR2111	a,b	Kelch repeat-containing F-box	**At3g27150**	Phosphate metabolism	-P	[12], [13]
NSI	miR156	h	SPL transcription factors	**At1g53160**	Vegetative phase change	-P -K	[12], [21], [54]
				**At2g33810**			
				**At3g15270**			
				**At5g43270**			
				**At1g27360**			
				**At1g27370**			
				**At1g69170**			
				**At2g42200**			
				**At3g57920**			
				**At5g50570**			
	miR160	a–c	Auxin response factors	**At2g28350−**	Root/Flower development		[21], [43]
				**At4g30080−**			
				**At1g77850−**			
	miR171	b,c	SCL transcription factors	**At2g45160−**	Root development		[45]
				**At3g60630−**			
				**At4g00150−**			
	miR780.1		Na+/H+ antiporter family	**At5g41610**	Sodium ion export		[35]
				At4g33260			
	miR826		Alkenyl hydroxalkyl producing 2	At4g03060−	Glucosinolate synthesis		[22], [55]
	miR829.1		*	*	*		
	miR829.2		AP2 domain ethylene response	At5g18560	Root development		[52], [55]
	miR839		*	*	*		
	miR842		Jacalin lectin family protein	At1g60130	Unknown		[22], [55]
				At1g57570			
				**At5g38550**			
	miR846		Jacalin lectin family protein	**At5g49850**	Unknown		[55]
				At2g25980			
				At5g49870			

Boldface letters indicate the previously validated targets. “*”indicates that no target was predicted. “+” and “−” indicate that up-regulation and down-regulation by N-starvation.

To establish the targets of one miRNA, it is necessary to find the corresponding cleaved products of its target transcripts. Recently, many miRNA targets were identified in rice and *A. thaliana* by degradome sequencing technology [22]–[24]. To confirm the predicted targets, the released *A. thaliana* degradome dataset was searched for potential cleavage sites of target transcripts. The new target of miR158, *At2g03220*, which encodes a fucosyltransferase, was cleaved in the miR158 recognition site. We found that miR775 was predicted to target genes coding fucosyltransferases responsible for transferring fucose groups. N starvation can leads to hexose accumulation in *Arabidopsis* [25]. Fucosyl residues have a role in the symbiotic interaction between legumes and *Rhizobium* species. Therefore, miR158 and miR775 may be involved in the symbiotic interaction in N limitation. In addition, the target cleavage products of miR826, miR863 and miR1888 were also identified (Table 2). However, the roles of these targets in N starvation cannot be predicted.

### Many NSS miRNAs are involved in P, S, and Cu homeostasis

**Figure 3:**
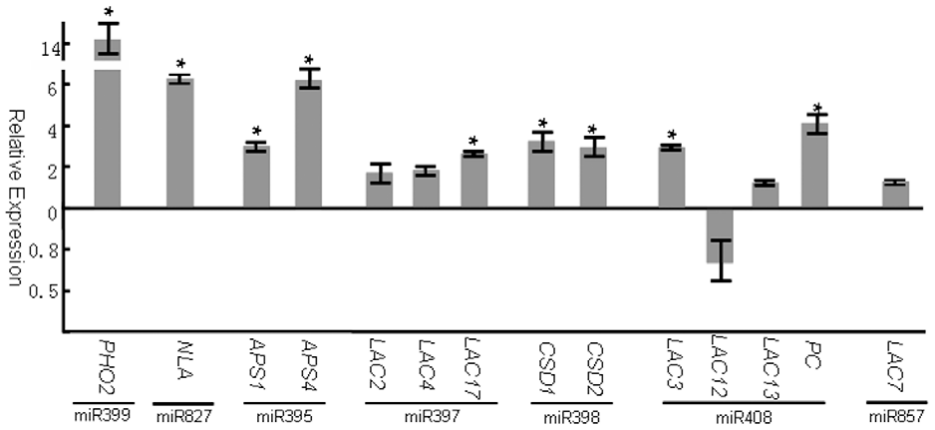
Expression of targets of different nutrient responsive miRNAs under N starvation conditions. Gene expression values shown are relative to the expression in plants grown under normal MS medium, for which the value is set to 1. Error bars indicate ± SE obtained from three biological repeats. Values marked by an asterisk are significantly different from the corresponding control value with Student's *t*-test (*p*<0.01; *n* = 3).

Several miRNAs have been identified to regulate nutrient metabolism [10], [11], [15], [17], [26]. We identified 23 NSS miRNAs, 9 of which directly take part in nutrient homeostasis (Table 2). Three miRNAs (miR399, miR827, and miR2111) were identified to function in P homeostasis. miR399 is specifically induced by P starvation and promotes phosphate uptake by cleavage of *PHO2* transcripts. *pho2* mutant plants showed phosphate-toxic phenotypes similar to miR399 over-expressing plants [11]. P starvation also simultaneously induced miR827 and miR2111 [12], [13], both of which were also clearly repressed by N limitation (Figure 3; Table 2). Very recently, miR827 was identified to function in phosphate absorption by targeting *NITROGEN LIMITATION ADAPTATION* (*NLA*) under N starvation [27]. When plants were subjected to N-deficient and P-sufficient conditions, *nla* mutant plants accumulated more phosphate than wild-type plants, displayed phosphate-toxic symptoms, and showed a decrease in anthocyanin synthesis. We revealed that both miR399 and miR827 were expressed at low levels under N-starvation conditions, with concomitant increase in expression of their targets, *PHO2* and *NLA* (Figure 3). This may be necessary for plants to prevent over-uptake of phosphate. miR2111 was confirmed to be induced by P starvation and to target a gene encoding a Kelch domain-containing F-box protein [12]. However, it is still unclear why miR2111 is suppressed by N starvation, but induced by P-starvation.

Both N and S are essential for plant growth and development, and the two assimilatory pathways are very similar and well coordinated. A deficiency in one element always results in suppression of the other pathway [28], [29]. miR395, a sulfate starvation-inducible miRNA, regulates sulfate accumulation and allocation in *Arabidopsis* leaves [10]. The over-expression of miR395 led to over-accumulation of sulfate in *Arabidopsis* leaves. In the present study, the abundance of miR395 decreased dramatically under N-starvation conditions, with moderate increases of its targets, *APS1* and *APS4* (Figure 3). Therefore, N starvation can reduce the accumulation of sulfate in leaves by repressing miR395. These results provide a new insight into the regulatory network between N and S assimilation pathways.

In addition to P or S starvation-induced miRNAs, Cu (copper) starvation-induced miRNAs were also down-regulated under N-starvation conditions. When plants were deprived of Cu, the abundance of miR397, miR398, miR408, and miR857 increased significantly. The target genes of these four miRNAs encode three kinds of copper-rich proteins (copper/zinc superoxide dismutase (CSD), plantacyanin, and laccase). Under Cu-starvation conditions, the up-regulation of these miRNAs can reduce the synthesis of copper-rich proteins, which facilitates the release of Cu to meet the Cu demand of plants. Under N starvation conditions, these four Cu-starvation-induced miRNAs were expressed at low levels and their target genes at high levels (Figure 3), suggesting that the Cu-starvation signal was inhibited by N deficiency. Similar results were also obtained in N starved maize roots [18], [30].

When subjected to low nutrient conditions, plants can sense and transmit nutrient deficiency signals. However, it is unclear how plants perceive and transmit low-N signals. miRNAs have been proved to be important signal molecules. Recent sequence analysis of miRNAs from *Brassica napus* [31] revealed that miR395 and miR399 are abundant in the phloem under low-sulfate and low-phosphate conditions, respectively. For example, miR399 was induced under low-phosphate conditions, and moves from shoots to roots in *Arabidopsis* [32]. In *B. napus*, miR395 is translocated through graft unions from scions to rootstocks under S-starvation conditions [33]. When exposed to Cu deficiency stress, rice and *Arabidopsis* over-accumulate miR397 and miR408 in the phloem. These miRNAs that accumulate in the phloem play roles in signal transduction. These nutrient deficiency-signaling molecules were significantly repressed by N starvation, which may decrease the amount of P, S, and Cu uptake. Deep sequencing analyses also suggested that miR169, miR395, and miR398 were expressed at low levels under P-deficient conditions [12]. Therefore, this miRNA-regulation mechanism is crucial for plants to maintain nutrient homeostasis and to adapt to nutrient deficiency stresses.

### Putative functions of N-starvation-induced (NSI) miRNAs

Two non-homologous NSI-miRNAs, miR842 and miR846, are processed from the same polycistron [34] and potentially target several *Jacalin Lectin* family genes. 5′-RACE experiments revealed that two jacalin lectin genes are targets of miR842 and miR846, respectively [35]. However, the functions of these *Jacalin Lectin* genes during N starvation remain unclear. The miR829 precursor can produce two different miR829 species, both of which were induced by N starvation. The putative targets of miR829.1 cannot be predicted; miR829.2 is predicted to target *PUCHI*, encoding an AP2 domain ethylene response factor, which is required for morphogenesis in the early lateral root primordium of *Arabidopsis* [36]. Thus, miR829 might regulate *Arabidopsis* root development under N starvation. The miR156 family contains three different mature miRNAs, all of which were up-regulated under N-deficient conditions. The fold-change of miR156h was the greatest, suggesting that there may be spatial and/or temporal differences in the functions of different miR156 members. In contrast, miR172 was repressed by N starvation (Table 2), which is consistency with the fact that miR156 negatively regulated the expression of miR172. miR156 and miR172 can prolong and promote the expression of juvenile vegetative traits in *Arabidopsis*, respectively [37], implying that N-starvation delays the transition of *Arabidopsis* from the vegetative to the reproductive phase by modulating the their abundance. miR780 targets a gene encoding a protein with sodium/hydrogen antiporter activity. The strong induction of miR780 could be responsible for the regulation of pH.

**Figure 4:**
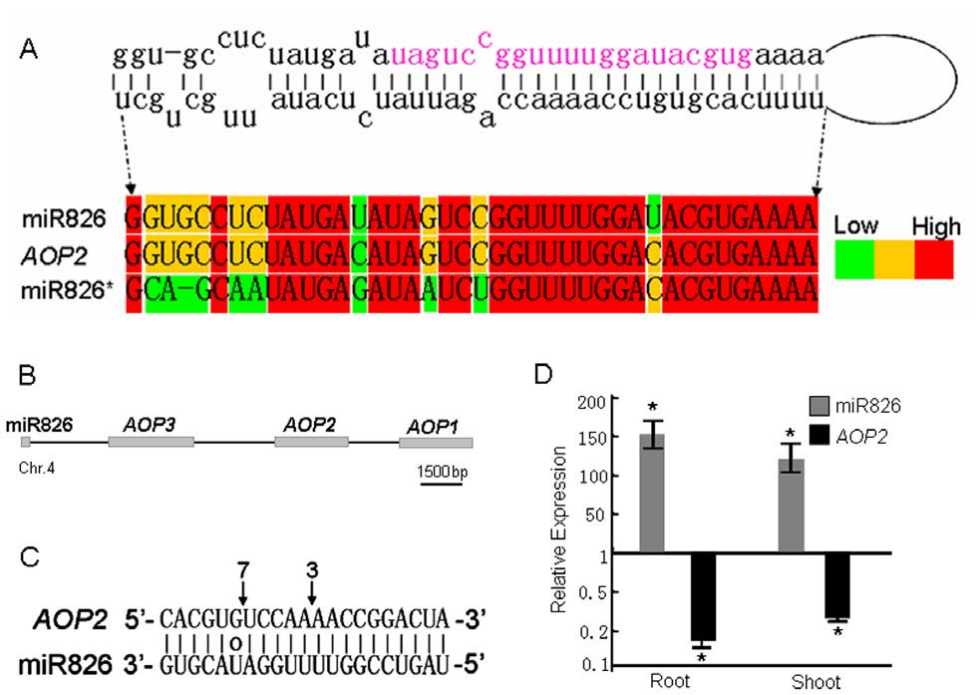
miR826 is a N-starvation-induced miRNA. (A)Comparative analysis of miR826 precursor and its target *AOP2* sequences. The sequence in pink indicates mature miR826 sequence. (B) The position of miR826, *AOP1*, *AOP2*, and *AOP3* genes in chromosome. (C) The cleavage sites of *AOP2* transcripts. (D) Expression of miR826 and *AOP2* in response to N starvation. Gene expression values shown are relative to the expression in plants grown under normal MS medium, for which the value is set to 1. Error bars indicate ± SE obtained from three biological repeats. Values marked by an asterisk are significantly different from the corresponding control value with Student's *t*-test (*p*<0.01; *n* = 3).

The abundance of miR826 significantly increased under N deficiency (Figure 1). miR826 was predicted to target the *AOP2* gene, which encodes a 2-oxoglutarate-dependent dioxygenase involved in glucosinolate biosynthesis. In *Arabidopsis*, miR826 is closely linked on the chromosome with its predicted target *AOP2*, as well as *AOP1* and *AOP3* (homologous genes of *AOP2*) (Figure 4B). Comparative genome analysis revealed that the miR826 precursor sequence is highly similar to that of the *AOP2* gene (Figure 4A). It is likely that the miR826 gene originated from duplication of its target gene [38]. Corresponding *AOP2* cleavage products matched with miR826 were also identified (Figure 4C). Interestingly, *AOP2* encodes a truncated and null-function protein due to a 5-bp deletion in the *AOP2* transcript of the Col ecotype [39]. The Cvi ecotype, which has normal *AOP2* transcripts, was used for further expression analysis. Consistent with the sequencing data, miR826 was dramatically induced in roots and shoots by N starvation. Correspondingly, N starvation significantly repressed the expression of its target, *AOP2* (Figure 4D). Glucosinolates are a group of plant secondary metabolites produced mainly in *Brassicas* and these compounds are rich in nitrogen and sulfur. Therefore, the suppression of *AOP2* by miR826 could decrease the production of glucosinolates, which would decrease the demand for N. However, further investigation is required to clarify the functions of miR826 during N starvation.

### Are miRNAs involved in root plasticity under N starvation?

N deficiency affects the morphogenesis of plant roots and often results in a stronger root system, such as more lateral roots. However, the mechanism underlying root development in response to N starvation is unclear. Recent studies suggested that miRNAs are regulators of root development and architecture [40]. For example, when Arabidopsis was subjected to nitrate treatment, miR167 and miR393 were induced to modulate root development [41], [42].

**Figure 5:**
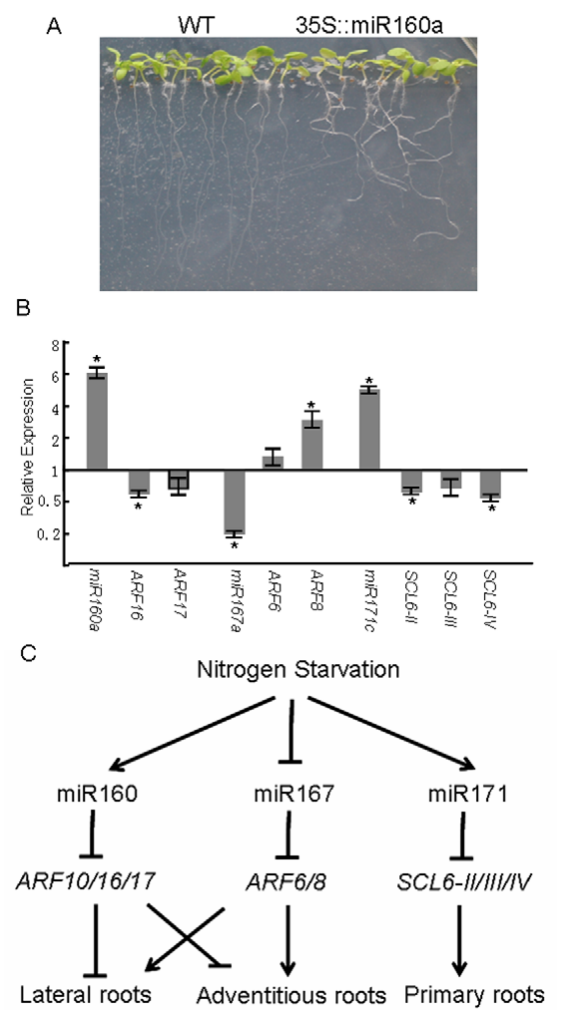
miR160, miR167, and miR171 are involved in development of root system under N starvation coditions. (A) More lateral roots in 35S::miR160a plants than WT (wild-type) plants. (B) Expression of miR160, miR167, miR171, and their targets under N starvation conditions. Gene expression values shown are relative to the expression in plants grown under normal MS medium, for which the value is set to 1. Error bars indicate ± SE obtained from three biological repeats. Values marked by an asterisk are significantly different from the corresponding control value with Student's *t*-test (*p*<0.01; *n* = 3). (C) A putative work model for miRNA-mediated root growth under N starvation conditions.

In the sequencing results, the abundance of miR160 increased 6-fold under N-deficient conditions, compared with that under N-sufficient conditions. The expression of miR160a and its targets was determined under N-deficient conditions. With the induction of miR160a, its targets *ARF16* and *ARF17* were down-regulated under N deficiency (Figure 5B). Studies suggested that miR160 controls lateral root formation by mediating regulation of *ARF16* [43]. To investigate whether the increased abundance of miR160 facilitates lateral root formation during N starvation, miR160a-overexpressing transgenic plants (35S::miR160a) were constructed. As expected, all miR160 over-expressing plants produced more lateral roots than wild type plants (Figure 5A). These results implied that N deficiency induced expression of miR160, which then mediated the cleavage of *ARF16* and promoted lateral root production in *Arabidopsis*.

miR167 takes part in the plasticity of roots by mediating the regulation of *ARF8* in response to N treatment [41]. The sequencing data showed that miR167 was repressed under N-deficient conditions. As expected, the inhibition of *ARF8* by miR167 was relieved (Figure 5B), which could promote lateral root outgrowth. In addition to the plasticity of lateral roots, miR160 and miR167 also play crucial roles in adventitious root development [44]. miR160 facilitates adventitious root outgrowth via repressing *ARF17*, whereas miR167 negatively regulates adventitious root initiation via cleavage of *ARF6* and *ARF8*. Therefore, both the increased miR160 and decreased miR167 favor root growth under N-starvation conditions.

Recent research showed that miR171 decreased primary root elongation by cleaving three *SCL6* genes [45], [46]. Under N-deficient conditions, the abundance of miR171 was three-fold higher than that under N-sufficient conditions. Quantitative RT-PCR analyses showed that the expression of miR171c was clearly up-regulated in the root under N starvation (Figure 5B). Correspondingly, the three targets of miR171 were down-regulated significantly by N starvation. It is very likely that induction of miR171 results in decreased expression of its targets (*SCL6-II*, *SCL6-III*, and *SCL6-IV*) (Figure 5B), which then suppresses the elongation of primary roots during N starvation in *Arabidopsis*.

miR160, miR167, and miR171 are involved in the signaling pathways triggering root system development. From these results, a conclusion was drawn that in response to N deficiency, plants may enhance their root systems by inducing expression of miR160 and decreasing those of miR167 and miR171 (Figure 5C).

### Novel miRNAs and their targtets

In addition to the known miRNAs, 20 novel miRNAs were also identified based on standard annotation criteria [47], nine of which were responsive to N starvation (Table 3). These new miRNAs were found in both N-deficient and N-sufficient samples. The read number of most novel miRNAs was much lower than that of the conserved miRNAs. This is consistent with the conclusion that non-conserved miRNAs are usually expressed at lower levels and with a spatial and temporal pattern. Precursors of these novel miRNAs were identified and their putative secondary hairpin structures were predicted (Table S4). The corresponding miRNA* sequences of four novel miRNAs (miRN01, miRN03, miRN06, miRN14) were found in the sequencing data (Table S4).

**Table 3:** Novel miRNAs identified from deep sequencing data.

miRNA	Sequence	Length	+N	−N	Fold(log2)(−N/+N)	Target	Target Annotation
miRN01	TGAGAGAAGGAATTAGATTCA	21	22	18	−0.3	At3g60830	Unknown
						At4g28760	
miRN02	CTTGAGGAGGTGTATAGAGGTTA	23	6	8	0.4	-	
miRN03	TTTTACTGCTACTTGTGTTCC	21	9	13	0.5	At3g09010	Protein kinase
miRN04	ATACTGAAGATGAAACTAGCT	21	6	6	0	**At1g75280**	Isoflavone reductase
miRN05	TAGTGTTTTTTATGGATCGTCTA	23	12	28	1.2	-	
miRN06	AAAGATGCAGATCATATGTCC	21	17	38	1.2	-	
miRN07	TGGCGAGGATGAATAATGCTAA	22	28	153	2.4	-	
miRN08	TTCGGTTCGGTTCGGTTCGGTTA	23	14	27	0.9	**At1g74930**	Chloroplast function
						**At1g67623**	
						**At1g26520**	
miRN09	TTGGTAGTGGATAAGGGGGCA	21	71	226	1.7	At1g80740	Chromomethylase 1
miRN10	TACAGAGTAGTCAAGCATGACC	22	21	26	0.3	-	
miRN11	TTGTCGATGTTTTTTTTACGGTA	23	13	13	0	-	
miRN12	TGGGGTATTGTTGGAGTTTATTA	23	9	7	−0.4	-	
miRN13	TTGACTGCATTAACTTGATCG	21	24	9	−1.4	**At1g26450**	Carbohydrate-binding protein
miRN14	TGACATCCAGATAGAAGCTTT	21	1145	82	−3.8	**At3g59210**	F-box family protein
						At1g58310	
						At5g41840	
						At3g59230	
						At4g00320	
						At3g59200	
miRN15	AGACACGGAGAAATCGGGAGATC	23	9	13	0.5	-	
miRN16	ACAGTGGTCATCTGGTGGGCT	21	8	22	1.5	-	
miRN17	AAAGAATCGTTGTTCAAGCTA	21	8	36	2.2	-	
miRN18	TTTGAATTTTTAGAGCATGTCCA	23	12	11	−0.1	-	
miRN19	TTAGTCGAATATGTTTTGGTTTA	23	5	6	0.3	-	
miRN20	TACAAGGAGTCAAGCATGACC	21	14	81	2.5	**At3g24610**	F-box family protein

Boldface letters indicate that the predicted target cleavages were identified from *Arabidopsis* degradome data.

“-” Indicates that no target was predicted. The miRNAs with a relative change ratio greater than 2 were underlined.

**Figure 6:**
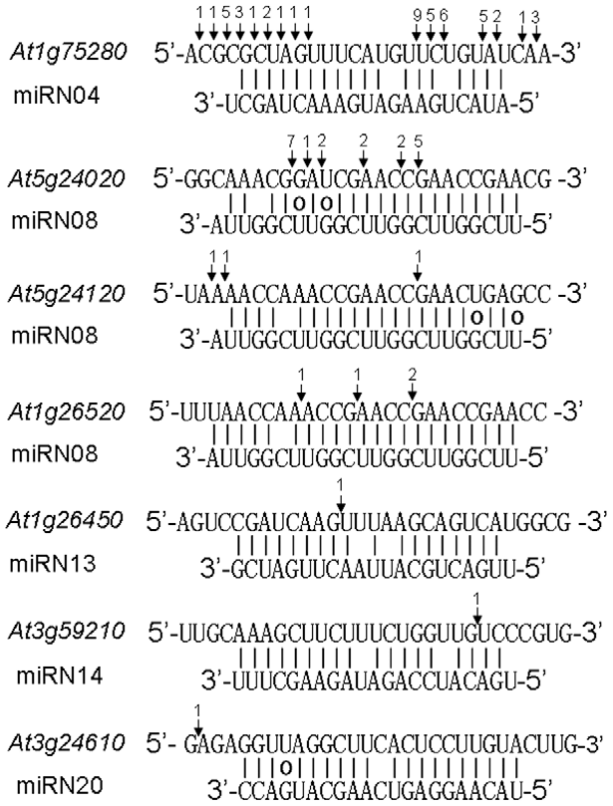
Identified targets of novel miRNAs from *Arabidopsis* degradome data. Vertical arrows indicate the target cleavage positions. The number indicates the number of corresponding cleavage products.

To explore the functions of these novel miRNAs, we predicted their potential target genes. However, the targets of only eight novel miRNAs were predicted with no predicted targets for the remainder (Table 3). To confirm the prediction results, we identified target cleavage sites from degradome data [22], [23]. Target gene cleavage products of five miRNAs (miRN04, miRN08, miRN13, miRN14, and miRN20) were identified (Figure 6). miRN04 targets an isoflavone reductase encoding gene, but its abundance was not affected by N starvation. Out of six predicted target genes for miRN08, corresponding cleavage of three target genes were found. *At1g26520* encodes a protein with unknown functions. The products of both *At5g24020* and *At5g24120* function in chloroplast. *At1g26450* is the potential targets of miRN13, which encode proteins located in membrane to bind carbohydrate. It is possible that the decrease of miRN13 facilitate energy supply of cells during N starvation. Both miRN14 and miRN20 target F-box protein encoding genes. P-starvation-induced miRNAs (miR399, miR827, and miR2111) are involved in ubiquitin-mediated protein degradation [11]–[13], [27] and all of them were repressed greatly by N starvation. Notably, the abundance of miRN14 decreased in response to N starvation, and its target genes also were involved in ubiquitin-mediated protein degradation. Therefore, posttranslational regulation of protein abundance may be crucial for the adaptation of plants to N starvation environment. However, further investigation is required to understand the functions of these novel miRNAs under N starvation conditions.

### Conclusion

Recently, several miRNAs were identified to be responsive to N limitation in *Arabidopsis*, which includes miR156, miR167, miR169, and miR398 [13], [17]. However, it is unclear how other plant miRNAs respond to N starvation. This study presented an extensive survey of the miRNAs showing differential expression in response to N starvation in *Arabidopsis*. These N-responsive miRNAs and their target genes are likely involved in the development or regulation of the adaptive response to N starvation. The identification of these N-starvation-responsive miRNAs provides new insights into the molecular mechanism of adaptation of plants to N deficiency.

Nutrient balance and homeostasis are crucial for plant growth and development [48]. The deficiency of one mineral element often affects absorption of other mineral elements. P, S, Cu-starvation-induced miRNAs were suppressed by N starvation, suggesting that these miRNAs may mediate the crosstalk among N, P, S, and Cu under N-starvation conditions. The mobility of these miRNAs means that they are candidate molecules for nutrient-deprivation signal transduction [13], [32]. Their up-regulation or down-regulation coordinates and balances the demands of different nutrients. Homeostasis of N in growing plants requires a sustained uptake of N into root cells. In most situations, adjustment of N acquisition by the roots according to the nutrient demands of the plant is hampered by the limiting and fluctuating availability of N in soil. Higher plants modulate their root uptake capacity to compensate for changes in external N concentrations. Our results suggested that changes in the expression levels of miR160, miR167, and miR171 may be important for the enhancement of plant root system under nitrogen deficiency.

Previously, most researches on nitrogen sensing focused on metabolism and morphological responses to the addition of nitrate [49]–[52]. Recently, experiments that focus on the switch from N-sufficient to -deficient conditions have received more attention [18], [25], [30]. Our work will help us to further understand the responses and signal transduction pathways for N starvation.

## Materials and Methods

### Plant materials and growth conditions

*Arabidopsis* (*A. thaliana* ecotype Columbia) wild-type plants were used to construct small RNAs libraries. Seeds were surface-sterilized with 20% (v/v) bleach and washed three times with distilled water before plating on MS medium (containing 0.8% agar). Three days after vernalization, plates with seeds were moved to a growth cabinet. For N-sufficient (+N) conditions, plants were grown on MS medium; for N-deficient (−N) conditions, plants were grown on modified MS medium (without NH_4_NO_3_ and with KCl instead of KNO_3_). For P-deficient (−P) conditions, plants were grown on modified MS medium in which PO_3_^3−^ was replaced by Cl^−^; for S-deficient (−S) conditions, plants were grown on modified MS medium in which SO_4_^2−^ was replaced by Cl^−^. Ten-day-old seedlings were used for RNA extraction.

### RNA preparation and small RNA sequencing

The whole seedlings grown in +N or −N medium were sampled to generate small RNA libraries. Total RNA was extracted using Trizol reagent (Invitrogen). Low molecular weight RNA was isolated from 200 µg total mRNAs by PEG8000/NaCl precipitation. Small RNAs in the size range of 20 to 30 nucleotides were purified from 15% denaturing polyacrylamide gels and ligated first with the 5′ RNA adaptor and then with the 3′ RNA adaptor. At each step, the ligated products were purified by electrophoretic separation on polyacrylamide gels. After first-strand synthesis and 18 cycles of PCR amplification, the final bands were purified on PAGE gels and submitted for sequencing. Sequencing was performed at the Beijing Genomics Institute (BGI).

### Computational analysis of sequencing data

The raw sequencing data were trimmed by removing adaptor sequences and mapped to the *Arabidopsis* genome (The Arabidopsis Information Resource (TAIR) release version 10; http://www.arabidopsis.org/). Reads perfectly matching those in the *Arabidopsis* genome, excluding those matching tRNAs, rRNAs, snRNA, and snoRNAs, were used for further analysis. *Arabidopsis* mature miRNAs and their precursors were retrieved from miRBase (version 14; http://www.mirbase.org).

### miRNA identification and target prediction

New miRNAs were identified as described by Fahlgren et al. [35]. WMD3 software package was used for the target prediction of miRNAs. The degradome data were from experiments described in Addo-Quaye et al. [23] and German et al. [22] (http://www.ncbi.nlm.nih.gov/geo/; GEO accession number GSE11007 and GSE11093).

### Generation of transgenic plants

The miR160a precursor sequence was inserted into the pOCA30 binary vector. The binary vector was then transformed into *Agrobacterium tumefaciens* strain GV3101. *Arabidopsis* was transformed by the floral-dip method. Transgenic plants were selected on MS medium supplemented with 50 µg/mL kanamycin.

### Gene expression analysis

Root samples were harvested separately from *Arabidopsis* and frozen in liquid nitrogen for storage at −80°C. Total RNA was isolated by Trizol reagent (Invitrogen) and digested by DNaseI (Fermentas).

Expression of miRNAs was detected by stem-loop RT-PCR. To produce miRNA-fused stem-loop cDNA, 0.5 µg total RNA was used for the reverse transcription with miRNA mature-sequence-specific stem-loop RT primers according to the stem-loop RT-PCR protocol [53]. For mRNA cDNA, 0.5 µg total RNA was reverse-transcribed using oligo(dT)18 primer according to the reverse transcription protocol (Fermentas). A 20-µl reaction mixture was used for the production of cDNA. After heat inactivation, a 1-µl aliquot was used as a template for real-time quantitative RT-PCR. An miRNA-specific primer and a universal primer were used to amplify miRNA-fused cDNA. Two specific primers were used to amplify each miRNA target gene. All primers used in this study are listed in Table S3. *Arabidopsis ACT2* (At3g18780) was used as an internal control for real-time RT-PCR. All quantitative RT-PCR analyses were performed using a SYBR Premix Ex Taq™ kit (TaKaRa) on a Roche LightCycler 480 real-time PCR machine, according to the manufacturer's instructions.

## Supporting Information

Figure S1 **Summary of deep sequencing data.**
(DOC)

Table S1 **Summary of miRNAs.**
(DOC)

Table S2 **Normalized abundances of miRNAs.**
(DOC)

Table S3 **Primers used in this study.**
(DOC)

Table S4 **Putative secondary structure of novel miRNA precursors.**
(TXT)
