# Supplementary material for: Identification of Nitrogen Starvation-Responsive MicroRNAs in Arabidopsis thaliana
Source: PLoS One. 2012 Nov 14;7(11):e48951. doi: 10.1371/journal.pone.0048951 (PMC3498362; doi:10.1371/journal.pone.0048951)

**Figure S1.** Summary of deep sequencing data. (A) Distribution of different length sequence reads in N-starvation and control samples. (B) Distribution of sRNAs in N-starvation and control samples.


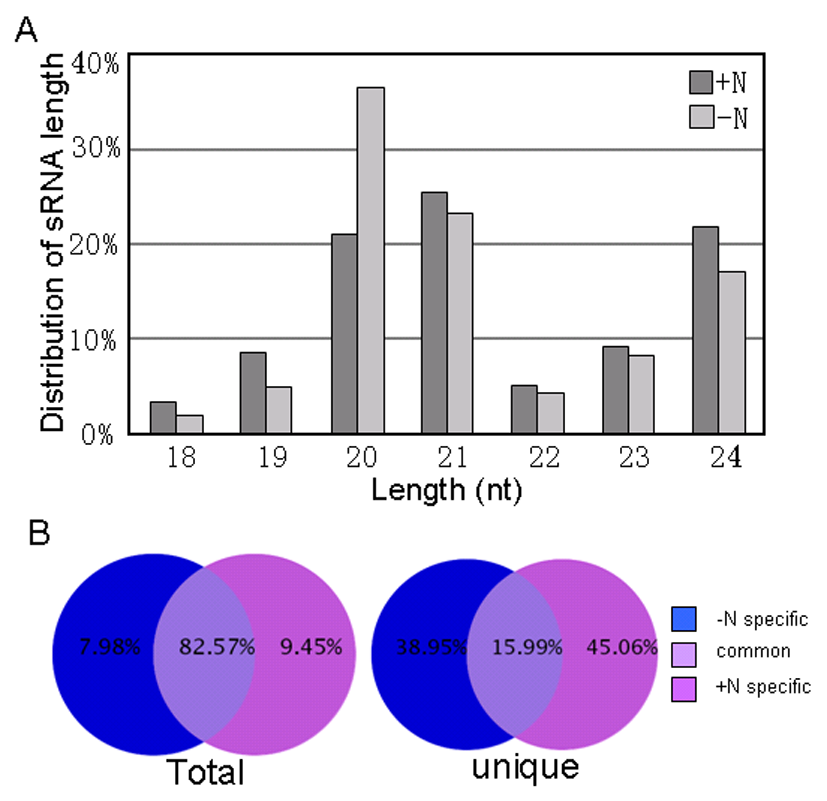

Supplement: Figure S1 — Summary of deep sequencing data. (DOC) [file pone.0048951.s001.doc]
