# Supplementary material for: Identification of Nitrogen Starvation-Responsive MicroRNAs in Arabidopsis thaliana
Source: PLoS One. 2012 Nov 14;7(11):e48951. doi: 10.1371/journal.pone.0048951 (PMC3498362; doi:10.1371/journal.pone.0048951)
Supplement: Table S1 — Summary of miRNAs. (DOC) [file pone.0048951.s002.doc]

| Table S1. Summary of miRNAs. | | | |
| --- | --- | --- | --- |
|  | +N | -N | Arabidopsis |
| unique | 143 | 132 | 162 |
|  | 88% | 81% | 100% |
| family | 93 | 84 | 112 |
|  | 83% | 75% | 100% |
