# Supplementary material for: Identification of Nitrogen Starvation-Responsive MicroRNAs in Arabidopsis thaliana
Source: PLoS One. 2012 Nov 14;7(11):e48951. doi: 10.1371/journal.pone.0048951 (PMC3498362; doi:10.1371/journal.pone.0048951)
Supplement: Table S2 — Normalized abundances of miRNAs. (DOC) [file pone.0048951.s003.doc]

| Table S2. Normalized abundances of miRNAs | | | |
| --- | --- | --- | --- |
| miR-name | N+ | N- | folds log_2_ (N-/N+) |
| ath-miR156a | 1402792.0 | 1692610.2 | 0.3 |
| ath-miR156b | 1402901.5 | 1692734.6 | 0.3 |
| ath-miR156c | 1402792.0 | 1692610.2 | 0.3 |
| ath-miR156d | 1453165.1 | 1717660.3 | 0.2 |
| ath-miR156e | 1401254.0 | 1690710.4 | 0.3 |
| ath-miR156f | 1401261.2 | 1690710.4 | 0.3 |
| ath-miR156g | 6095.2 | 8236.0 | 0.4 |
| ath-miR156h | 476.2 | 3028.6 | 2.7 |
| ath-miR157a | 1287368.0 | 1236057.5 | -0.1 |
| ath-miR157b | 1287370.0 | 1236059.4 | -0.1 |
| ath-miR157c | 1292923.0 | 1234726.5 | -0.1 |
| ath-miR157d | 49591.2 | 23070.6 | -1.1 |
| ath-miR158a | 331232.7 | 115256.0 | -1.5 |
| ath-miR158b | 3383.8 | 2850.7 | -0.2 |
| ath-miR159a | 1589.7 | 1608.1 | 0.0 |
| ath-miR159b | 584.6 | 534.7 | -0.1 |
| ath-miR159c | 294.4 | 311.1 | 0.1 |
| ath-miR160a | 455.5 | 2744.7 | 2.6 |
| ath-miR160b | 282.0 | 1941.6 | 2.8 |
| ath-miR160c | 457.6 | 2745.6 | 2.6 |
| ath-miR161.1 | 33894.0 | 31919.1 | -0.1 |
| ath-miR161.2 | 30500.9 | 18067.4 | -0.8 |
| ath-miR162a | 1632.0 | 1214.3 | -0.4 |
| ath-miR162b | 1634.1 | 1217.3 | -0.4 |
| ath-miR163 | 344.0 | 406.4 | 0.2 |
| ath-miR164a | 19514.8 | 20717.8 | 0.1 |
| ath-miR164b | 19622.3 | 20790.7 | 0.1 |
| ath-miR164c | 2187.7 | 1765.6 | -0.3 |
| ath-miR165a | 13390.7 | 32758.2 | 1.3 |
| ath-miR165b | 13087.0 | 32358.6 | 1.3 |
| ath-miR166a | 106697.9 | 127168.1 | 0.3 |
| ath-miR166b | 101284.4 | 119487.3 | 0.2 |
| ath-miR166c | 101405.3 | 119616.6 | 0.2 |
| ath-miR166d | 101406.3 | 119617.6 | 0.2 |
| ath-miR166e | 101302.0 | 119567.0 | 0.2 |
| ath-miR166f | 101299.9 | 119565.1 | 0.2 |
| ath-miR166g | 101421.8 | 119695.3 | 0.2 |
| ath-miR167a | 425863.1 | 262503.1 | -0.7 |
| ath-miR167b | 426303.2 | 262698.6 | -0.7 |
| ath-miR167c | 859.4 | 3486.5 | 2.0 |
| ath-miR167d | 3540.8 | 2262.4 | -0.6 |
| ath-miR168a | 57061.3 | 67759.4 | 0.2 |
| ath-miR168b | 57011.7 | 67702.0 | 0.2 |
| ath-miR169a | 2703.1 | 931.4 | -1.5 |
| ath-miR169b | 259.3 | 65.1 | -2.0 |
| ath-miR169c | 213.8 | 50.6 | -2.1 |
| ath-miR169d | 738.5 | 1065.6 | 0.5 |
| ath-miR169e | 738.5 | 1065.6 | 0.5 |
| ath-miR169f | 693.1 | 1057.8 | 0.6 |
| ath-miR169g | 687.9 | 1048.1 | 0.6 |
| ath-miR169h | 223.1 | 13.6 | -4.0 |
| ath-miR169i | 671.4 | 56.4 | -3.6 |
| ath-miR169j | 664.2 | 53.5 | -3.6 |
| ath-miR169k | 224.1 | 13.6 | -4.0 |
| ath-miR169l | 662.1 | 53.5 | -3.6 |
| ath-miR169m | 240.7 | 14.6 | -4.0 |
| ath-miR169n | 664.2 | 53.5 | -3.6 |
| ath-miR170 | 28.9 | 41.8 | 0.5 |
| ath-miR171a | 595.0 | 201.3 | -1.6 |
| ath-miR171b | 79.5 | 245.2 | 1.6 |
| ath-miR171c | 80.6 | 245.2 | 1.6 |
| ath-miR172a | 11183.4 | 2079.7 | -2.4 |
| ath-miR172b | 11182.3 | 2079.7 | -2.4 |
| ath-miR172c | 72.3 | 127.4 | 0.8 |
| ath-miR172d | 72.3 | 127.4 | 0.8 |
| ath-miR172e | 283.0 | 514.3 | 0.9 |
| ath-miR173 | 16676.4 | 22590.4 | 0.4 |
| ath-miR1886.1 | 30.0 | 21.4 | -0.5 |
| ath-miR1886.2 | 512.3 | 396.7 | -0.4 |
| ath-miR1886.3 | 12.4 | 2.9 | -2.1 |
| ath-miR1888 | 192.1 | 20.4 | -3.2 |
| ath-miR2111a | 200.4 | 19.4 | -3.4 |
| ath-miR2111b | 200.4 | 19.4 | -3.4 |
| ath-miR319a | 119.8 | 91.4 | -0.4 |
| ath-miR319b | 66.1 | 89.4 | 0.4 |
| ath-miR319c | 25.8 | 17.5 | -0.6 |
| ath-miR390a | 11709.1 | 14016.0 | 0.3 |
| ath-miR390b | 11703.9 | 14003.4 | 0.3 |
| ath-miR391 | 1325.2 | 1649.9 | 0.3 |
| ath-miR393a | 53.7 | 37.9 | -0.5 |
| ath-miR393b | 53.7 | 37.9 | -0.5 |
| ath-miR394a | 246.9 | 258.6 | 0.1 |
| ath-miR394b | 246.9 | 258.6 | 0.1 |
| ath-miR395a | 45.4 | 6.8 | -2.7 |
| ath-miR395b | 77.5 | 1.0 | -6.3 |
| ath-miR395c | 77.5 | 1.0 | -6.3 |
| ath-miR395d | 45.4 | 6.8 | -2.7 |
| ath-miR395e | 43.4 | 6.8 | -2.7 |
| ath-miR395f | 77.5 | 1.0 | -6.3 |
| ath-miR396a | 4167.8 | 5193.8 | 0.3 |
| ath-miR396b | 3119.4 | 3820.0 | 0.3 |
| ath-miR397a | 93.0 | 25.3 | -1.9 |
| ath-miR397b | 785.0 | 22.4 | -5.1 |
| ath-miR398b | 48.5 | 1.9 | -4.6 |
| ath-miR398c | 48.5 | 1.9 | -4.6 |
| ath-miR399a | 162.2 | 0.1 | -10.7 |
| ath-miR399b | 1229.2 | 1.0 | -10.3 |
| ath-miR399c | 1207.5 | 1.0 | -10.3 |
| ath-miR399d | 110.5 | 0.1 | -10.1 |
| ath-miR399e | 40.3 | 0.1 | -8.7 |
| ath-miR399f | 363.6 | 0.1 | -11.8 |
| ath-miR400 | 472.0 | 262.5 | -0.8 |
| ath-miR402 | 829.4 | 942.1 | 0.2 |
| ath-miR403 | 20263.7 | 20248.2 | 0.0 |
| ath-miR408 | 731.3 | 189.6 | -1.9 |
| ath-miR447a | 289.2 | 127.4 | -1.2 |
| ath-miR447b | 289.2 | 127.4 | -1.2 |
| ath-miR773 | 45.4 | 78.8 | 0.8 |
| ath-miR775 | 4600.6 | 1468.1 | -1.6 |
| ath-miR777 | 72.3 | 97.2 | 0.4 |
| ath-miR779.2 | 9.3 | 10.7 | 0.2 |
| ath-miR780.1 | 4.1 | 23.3 | 2.5 |
| ath-miR780.2 | 9.3 | 17.5 | 0.9 |
| ath-miR781 | 23.8 | 11.7 | -1.0 |
| ath-miR783 | 51.6 | 11.7 | -2.1 |
| ath-miR822 | 34604.7 | 21123.2 | -0.7 |
| ath-miR823 | 353.3 | 838.1 | 1.2 |
| ath-miR824 | 558.8 | 1129.8 | 1.0 |
| ath-miR825 | 313.0 | 34.0 | -3.2 |
| ath-miR826 | 1.0 | 87.5 | 6.4 |
| ath-miR827 | 360.5 | 12.6 | -4.8 |
| ath-miR829.1 | 658.0 | 3577.9 | 2.4 |
| ath-miR829.2 | 3.1 | 71.9 | 4.5 |
| ath-miR830 | 18.6 | 8.8 | -1.1 |
| ath-miR833-3p | 14.5 | 10.7 | -0.4 |
| ath-miR833-5p | 66.1 | 34.0 | -1.0 |
| ath-miR835-3p | 15.5 | 9.7 | -0.7 |
| ath-miR837-3p | 582.6 | 1244.5 | 1.1 |
| ath-miR837-5p | 43.4 | 128.3 | 1.6 |
| ath-miR839 | 6.2 | 72.9 | 3.6 |
| ath-miR841 | 179.7 | 9.7 | -4.2 |
| ath-miR842 | 72.3 | 393.8 | 2.4 |
| ath-miR843 | 166.3 | 86.5 | -0.9 |
| ath-miR844 | 23.8 | 18.5 | -0.4 |
| ath-miR845a | 7.2 | 14.6 | 1.0 |
| ath-miR846 | 866.6 | 3602.2 | 2.1 |
| ath-miR848 | 550.5 | 481.3 | -0.2 |
| ath-miR849 | 26.9 | 1.0 | -4.8 |
| ath-miR850 | 233.4 | 3.9 | -5.9 |
| ath-miR851-3p | 13.4 | 1.9 | -2.8 |
| ath-miR852 | 241.7 | 137.1 | -0.8 |
| ath-miR854a | 23.8 | 2.9 | -3.0 |
| ath-miR854b | 23.8 | 2.9 | -3.0 |
| ath-miR854c | 23.8 | 2.9 | -3.0 |
| ath-miR854d | 23.8 | 2.9 | -3.0 |
| ath-miR857 | 212.8 | 5.8 | -5.2 |
| ath-miR858 | 16.5 | 25.3 | 0.6 |
| ath-miR860 | 104.3 | 82.6 | -0.3 |
| ath-miR861-5p | 20.7 | 3.9 | -2.4 |
| ath-miR862-5p | 19.6 | 9.7 | -1.0 |
| ath-miR863-3p | 2818.8 | 93.3 | -4.9 |
| ath-miR863-5p | 18.6 | 0.1 | -7.5 |
| ath-miR864-3p | 11.4 | 4.9 | -1.2 |
| ath-miR864-5p | 127.0 | 78.8 | -0.7 |
| ath-miR865-5p | 11.4 | 17.5 | 0.6 |
| ath-miR866-5p | 103.3 | 268.3 | 1.4 |
| ath-miR869.2 | 1126.9 | 567.8 | -1.0 |
