# Supplementary material for: Identification of Nitrogen Starvation-Responsive MicroRNAs in Arabidopsis thaliana
Source: PLoS One. 2012 Nov 14;7(11):e48951. doi: 10.1371/journal.pone.0048951 (PMC3498362; doi:10.1371/journal.pone.0048951)
Supplement: Table S3 — Primers used in this study. (DOC) [file pone.0048951.s004.doc]

Table S3. Primers used in this study.

qSCL6-II-F: CTTCTACCACCACCACGCTGTC

qSCL6-II-R: TATTGTTATCATCACCGGCGGT

qSCL6-III-F: CAGAGAAGCTCCATGGACGAGT

qSCL6-III-R: GACGAAAGGGCTTGGACTTCTA

qSCL6-IV-F: TTCTTTCTCGTCTTCCTTCCCC

qSCL6-IV-F: GCGGGAACTTCTTCCTCTTCTT

qARF6-F:CAAAGTTTAGCAGCTACCACGA

qARF6-R :ACGTCGTTCTCTCGGTCAAC

qARF8-F :TTTGCTATCGAAGGGTTGTTG

qARF8-R :CATGGGTCATCACCAAGGA

qARF16-F:CGTTAAGCTCTGTTCTGGAC

qARF16-R:AGTAATGGTGAAGATCCGAAG

qARF17-F:GCACCTGATCCAAGTCCTTC

qARF17-R:GGTGAATAGCTGGGGAGGAT

qPHO2-F :CCCCTTTGAAGTTTATCCAACTGG

qPHO2-R:AGGTGAGCCAACTGAGGACTCC

qAOP2-F:AGAGGACAAGATACACAGCAGCA

qAOP2-R:AAGTCGCGGTAATCAAAAGGTC

qNLA-F: ACAATTGTTCTCGTGAATGCCC

qNLA-R: GAGCATGCTCGTTAAACCATCC

qPC-F: GGCCAAGGGAAGAGGCAGTGC

qPC-R: ACCGACCGTGTACGTTGCAGC

qLAC12-F: AGAGACGCCGGTGAAGAGGCT

qLAC12-R: CTTCGAGCGTAGGCCCCGGA

qLAC13-F: AACGCCGGTGAAGAGGCTGT

qLAC13-R: AGGGAATCGCCGTTCCTCACCT

qLAC3-F: TCGCTTTCCTCGCTTCTGCTGA

qLAC3-R: ACCACAAGCGTTGGACCAGGGT

qLAC7-F: TGCCTCCATTGTGGAACACACCT

qLAC7-R: TGGTCCAGGCAGGCTTCCGT

qLAC2-F: ACTGATGGTGAAACCTGGAAAGACG

qLAC2-R: CGCTCCTACGACCGTCAATGTATGA

qLAC4-F: ACGGACACCCAGGCCCAGTT

qLAC4-R: ACCGTGAAAATATGGCCGGCGA

qLAC17-F: ACGATAAACGGGCTTCCTGGTCCA

qLAC17-R: ACCGTGTGATTTGCGATGCTGA

qCSD1-F: CAAGCACTTGATTCTTTCCAAAGGG

qCSD1-R: ACACCATCGCCTTCCTGGGTGA

qCSD2-F: ACATGACACACGGAGCTCCAGA

qCSD2-R: TTTCTGCCACGCCATCGGCA

qAPS1-F: AGGCTGGACAAGTCCACTCGG

qAPS1- R: GCCGTCGTCAAGACGTAGCGA

qAPS4-F: AGCGAAGGCTGGGCAAGTCC

qAPS4-R: AGCCGTCTTCGAGCCGGAAC

qACT2-F:TGTGCCAATCTACGAGGGTTT

qACT2-R:TTTCCCGCTCTGCTGTTGT

miR826-RT:GTCGTATCCAGTGCAGGGTCCGAGGTATTCGCACTGGATACGACCACGTA

miR826-F:GCAGCCTAGTCCGGTTTTGGA

miR169a-RT:GTCGTATCCAGTGCAGGGTCCGAGGTATTCGCACTGGATACGACTCGGCA

miR169bc-RT:GTCGTATCCAGTGCAGGGTCCGAGGTATTCGCACTGGATACGACCCGGCA

miR169d-g-RT:GTCGTATCCAGTGCAGGGTCCGAGGTATTCGCACTGGATACGACCGGCAA

miR169h-n-RT:GTCGTATCCAGTGCAGGGTCCGAGGTATTCGCACTGGATACGACCAGGCA

miR169abc-F: GCAGCCAGCCAAGGATGACT

miR169d-g-F: GCAGCGTGAGCCAAGGATGAC

miR169h-n-F: GCAGCGTAGCCAAGGATGACT

miR160a-RT: GTCGTATCCAGTGCAGGGTCCGAGGTATTCGCACTGGATACGACTGGCATA

miR160a-F: GCATGCTGCCTGGCTCCCTGT

miR167a-RT: GTCGTATCCAGTGCAGGGTCCGAGGTATTCGCACTGGATACGACTAGATC

miR167a-F: GCAGCCTGAAGCTGCCAGCAT

miR171c-RT: GTCGTATCCAGTGCAGGGTCCGAGGTATTCGCACTGGATACGACCGTGAT

miR171c-F: GCATGCTTGAGCCGTGCCAAT

Universal: GTGCAGGGTCCGAGGT
